# Emergence of splits and collective turns in pigeon flocks under predation

**DOI:** 10.1098/rsos.211898

**Published:** 2022-02-23

**Authors:** Marina Papadopoulou, Hanno Hildenbrandt, Daniel W. E. Sankey, Steven J. Portugal, Charlotte K. Hemelrijk

**Affiliations:** ^1^ Groningen Institute for Evolutionary Life Sciences, University of Groningen, Groningen, The Netherlands; ^2^ Centre for Ecology and Conservation, University of Exeter, Penryn, UK; ^3^ Department of Biological Sciences, School of Life and Environmental Sciences, Royal Holloway University of London, Egham, UK

**Keywords:** collective behaviour, escape patterns, self-organization, flocking, pigeon

## Abstract

Complex patterns of collective behaviour may emerge through self-organization, from local interactions among individuals in a group. To understand what behavioural rules underlie these patterns, computational models are often necessary. These rules have not yet been systematically studied for bird flocks under predation. Here, we study airborne flocks of homing pigeons attacked by a robotic falcon, combining empirical data with a species-specific computational model of collective escape. By analysing GPS trajectories of flocking individuals, we identify two new patterns of collective escape: early splits and collective turns, occurring even at large distances from the predator. To examine their formation, we extend an agent-based model of pigeons with a ‘discrete’ escape manoeuvre by a single initiator, namely a sudden turn interrupting the continuous coordinated motion of the group. Both splits and collective turns emerge from this rule. Their relative frequency depends on the angular velocity and position of the initiator in the flock: sharp turns by individuals at the periphery lead to more splits than collective turns. We confirm this association in the empirical data. Our study highlights the importance of discrete and uncoordinated manoeuvres in the collective escape of bird flocks and advocates the systematic study of their patterns across species.

## 1. Introduction

Patterns of collective escape of animals are some of the most complex and mesmerizing displays in nature: when a moving group is under attack by a predator, the group changes its shape and internal structure rapidly [1–4]. These patterns often confuse the predator and increase the prey's survival [5–8]. To counteract this, predators may attempt to split up their target groups [9,10]; for instance, an avian predator may attack an airborne flock several times [11,12], while gaining altitude in order to dive and intersect it at high speed (a hunting strategy called ‘stoop’) [13]. The anti-predator effect of aggregating is important in the evolution of group-living, particularly for species which are regularly preyed upon [7,14]. Because of the complexity of patterns of collective escape, however, the specifics concerning individual behaviour and coordination among group members when under attack are not so well understood.

Computational models are valuable for understanding the processes that underlie such complex patterns [15–17]. These underlying processes comprise specific behavioural rules that control the motion of individuals. To identify these rules, collective behaviour is often studied in models that are based on self-organization [18–20]. In these models, collective patterns emerge from local interactions of individuals, specifically from coordination among group members by attraction to, alignment with and repulsion from, their closest neighbours [21–25].

These rules of coordination have been similarly modelled across different taxonomic groups, for instance insect swarms, fish schools and bird flocks [23,26–29]. However, empirical data show that species may differ in their specifics of coordination [30–35]. In bird flocks, European starlings (*Sturnus vulgaris*) coordinate with their seven closest neighbours (referred to as ‘topological neighbours' [30]), jackdaws (*Corvus monedula*) with their mating partner and three other topological neighbours [33,36] and chimney swifts (*Chaetura pelagica*) with all individuals within a specific distance (referred to as ‘metric neighbours’, [32]). The number of interaction partners may even depend on the ecological context, as found in jackdaws during roosting and mobbing flights [32,37]. Moreover, the collective pattern may also be affected by the specifics of locomotion, for instance flight versus swimming or species-specific differences in flight modes [25,38]. Because of this variation, the rules of motion and coordination at the individual level of computational models can be adjusted to empirical data in order to study what patterns of collective motion emerge [24,39–43]. Species-specific models have, however, rarely been applied to bird flocks under predation [44].

Whether patterns of collective escape differ among species is not yet known; given the unpredictability and rarity of predator attacks, as well as the difficulty of simultaneously tracking both predator and prey, studying collective escape in the field is challenging. Complete trajectories of airborne birds in flocks under predation have only recently been collected with a newly developed, remotely controlled robotic-falcon (referred to as ‘RobotFalcon’ [45]) attacking flocks of homing pigeons (*Columba livia*) [46]. In the present study, we combine these quantitative data [46] with a species-specific computational model (HoPE—Homing Pigeons Escape, [47]) to investigate what behavioural rules underlie the patterns of collective escape in pigeons.

Patterns of collective escape in bird flocks have been extensively described only for large flocks of European starlings [2] (see also [48–50]). Their patterns include, among others: the agitation wave (dark bands moving over the flock away from the predator), flash expansion (where prey flees radially away from the predator), vacuole (where prey surrounds the predator) and splitting of sub-flocks [2,51]. Here, we identify two new patterns of collective escape in pigeons: namely collective turns (during which the whole flock changes its heading) and early splitting of sub-flocks (splits that occur also at large distance from the predator, see also [46]). Surprisingly, collective turns have so far been neglected in prior studies of collective escape in bird flocks [2].

**Figure 1.**
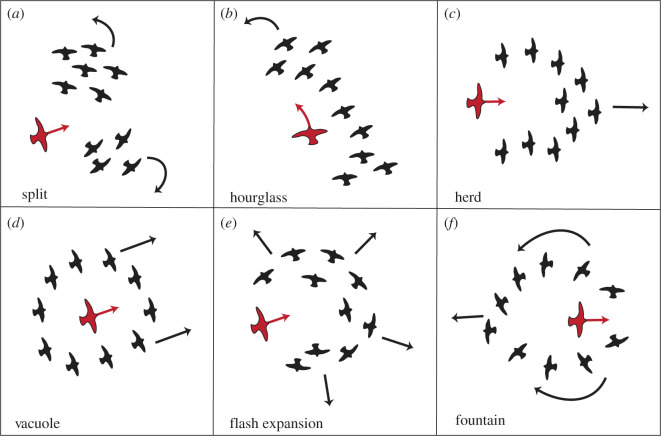
Patterns of collective escape generated by the computational model of fish schools by Inada *et al*. [26], adjusted to small flocks of 10 individuals. The black bird figures represent the prey and the larger red figure the predator.

To understand how these patterns arise, we need a new computational model since neither early splits nor collective turns have been reported in previous models of collective escape. In most models, individuals have only a single rule of escape, usually a continuous tendency to turn away from the predator while coordinating with their neighbours [26,47,52]. Remarkably, this single rule has led to many patterns in a model of fish schools [26] (figure 1), for instance the vacuole, the hourglass [9] and the split (in close vicinity to the predator). Some collective patterns were, however, not generated, for instance the agitation wave [53–55].

Agitation waves emerged in a computational model of starlings only through a fixed escape manoeuvre, which was copied by close-by neighbours and thus propagated through the group [44]. The manoeuvre was a zigzag-like turn, which involved banking while turning, resulting in a larger area of each individual's wings being exposed to the observer. This creates the visual effect of dark bands travelling over the flock [44,51]. The zigzag motion is an example of a ‘discrete’ escape manoeuvre, discrete meaning that a sharp change in heading or speed interrupts the continuous, coordinated motion of the group members [44,54,55]. Such manoeuvres are found in escaping dunlins (*Calidris alpina*) and are known to underlie escape waves in other species (e.g. U-turns in fish) [49,56–58]. Empirical data further suggest that the manoeuvres may not be fixed but varying and unpredictable in terms of turning angle and speed (referred to as ‘protean’ movement) to increase the complexity of the escape path of a prey [58–60].

To generate the early splits and collective turns (at large distance from the predator) that we see in pigeon flocks, we extend an agent-based model of pigeons' collective escape [31,47,61] with a new escape manoeuvre that is discrete, variable and uncoordinated. We give each individual a probability to turn away from the predator without coordinating with its neighbours. In order to adjust the escape rule to pigeons, we analyse their turning when they are under attack by the RobotFalcon. We uniquely sample each escape manoeuvre in our model from distributions of turning angle and duration parametrized to our empirical data. The probability to manoeuvre depends on each individual's distance to the predator and on a unique tendency to escape (according to empirical data of escape initiation [62,63]). We investigate whether both patterns of collective escape may arise from the same rule of escape at the individual level.

We show that both splitting and collective turning emerge in our model from a single escape manoeuvre. Based on theories of turning propagation [36,64,65] and information transfer [36,66], we expect collective turns to emerge more frequently when initiators: (i) are positioned more centrally in the group (directional information can reach the edges of the group faster), (ii) are turning slower and (iii) turn towards the group's centroid (making it easier for flock-mates to follow). We test these hypotheses in both our model and empirical data**.**

## 2. Methods

### 2.1. Empirical data

We analysed the GPS data of flocks of homing pigeons, collected by Sankey *et al.* [46]. The data were recorded during a short period immediately after the flocks' release using GPS loggers with a 5 Hz sampling frequency (QStarz BT-Q1300ST, Düsseldorf, Germany; see also [67] for a spatial resolution analysis). Our GPS data comprise preprocessed two-dimensional tracks of pigeons (the vertical positions were excluded because of inconsistent resolution [68]) from 43 groups of several sizes (namely of 8, 10, 27 and 34 pigeons). To study their collective escape, some of the pigeon flocks were attacked by a remotely controlled robotic predator (the RobotFalcon, mounted with an identical GPS logger), while other flocks were left to perform their homing flights uninterrupted (control data, no RobotFalcon) [46].

More specifically, the RobotFalcon resembled a male peregrine falcon (*Falco peregrinus*) in both appearance and motion [69], and flew with propellers mounted on the wings. The RobotFalcon is steered by a trained pilot (using goggles that receive images in real time from a First-Person View camera positioned on the head of the RobotFalcon) in a way that imitates the hunting behaviour of a real falcon during direct pursuit [46]. Where possible, the RobotFalcon attacked the flock by performing a stoop, sharply decreasing the distance between the falcon and the flock [69]. During each field experiment, a flock was released from a basket on the ground while the RobotFalcon was airborne (at approx. 5 m altitude) and approaching from approximately 50 m behind it. All flocks took flight immediately after release and were chased by the RobotFalcon until leaving the release site (a defined radius of 500 m). Thus, a flight in our dataset may consist of several instances of collective escape by a flock. For more details on the RobotFalcon, experimental set-up and preprocessing of GPS data, see Sankey *et al*. [46].

#### 2.1.1. Measuring patterns of collective escape

We first identified the patterns of collective escape in pigeons through video observations. In detail, we visualized the GPS data of flocks attacked by the RobotFalcon (see electronic supplementary material, video S1 for an example) and noted two patterns: collective turns and splits. To further study these patterns, we followed a quantitative approach.

**Figure 2.**
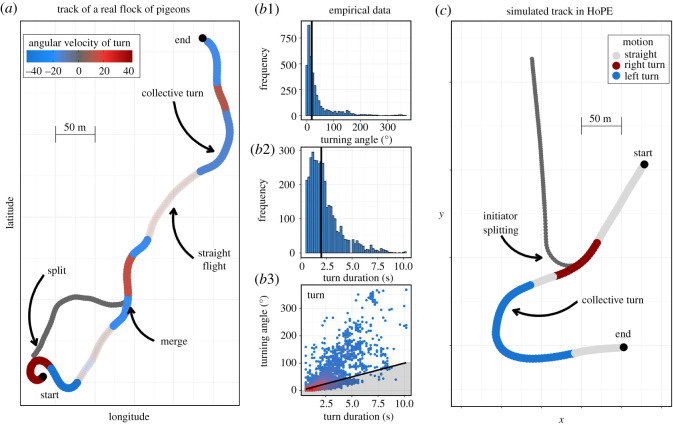
(*a*) Track of the average position of homing pigeons in a real flock starting a homing flight while being attacked by the RobotFalcon (GPS data from Sankey *et al*. [46]). A single individual splits off and subsequently returns to the group (merge). The colour shows the angular velocity of each collective turn (trajectory parts with angular velocity lower than 10° s^−1^ are considered straight flight and are coloured grey). (*b*) Turns of individual pigeons under attack, extracted from the tracks in the empirical data. The vertical black lines mark the median of each distribution. Turns of angular velocity at the top 2% of the distribution are omitted (see electronic supplementary material, figure S2 for the full distributions). These distributions are used to parametrize the escape manoeuvres of individuals in our model (the ‘initiators’ of a collective escape). (*b*1) Frequency distribution of the absolute angle turned (in degrees). (*b*2) Frequency distribution of the duration of each turn (in seconds). (*b*3) Turning angle versus duration of turn (angular velocity). Each point corresponds to the turn of one individual. The solid line shows the threshold we used to distinguish between turns and straight flight (angular velocity of 10° s^−1^). (*c*) Simulated tracks of a flock in the HoPE model. Two individuals consecutively perform an escape manoeuvre (sampled from distributions similar to *b*1 and *b*2). The first initiator (turning to the right relative to the flock's flying direction) splits from the group. From the turn of the second initiator (left turn), a collective turn emerges.

We quantified collective turning by first building the trajectory of each flock from the average position of all flock members across each flight. We then divided the trajectory in intervals in which the flock's angular velocity had the same sign (continuous turning to the left or to the right). Over each interval, we measured the cumulative change of heading. Small changes of heading were considered proxies for straight motion of the flock (average angular velocity less than 10° s^−1^, black lines in figure 2*b*3 and electronic supplementary material, figure S1). For each flight, we calculated the rate of turning (turns per second), the turning angle per second, and the proportion of time spent turning (for details see our electronic supplementary material). We tested whether these measurements differ between flights with and without the RobotFalcon, and ensured that our angular velocity threshold does not alter our conclusions (electronic supplementary material, table S1).

We determined a splitting event based on the distance between parts of a flock. The group with the larger number of individuals is referred to as the *‘*main flock’. We adapted the definition of splitting by Sankey *et al.* [46] by focusing on the collective [2] rather than individual behaviour. Specifically, an individual was considered to be split from the main flock if it has, for at least 2 s, no member of the main flock within a distance of 10 m [46]. From the perspective of the group, split events occur when flock size decreases. Thus, when several individuals were splitting from their group simultaneously, forming a sub-flock*,* we counted it as a single case of a split (instead of as many as the size of the sub-flock, as done in [46]). We calculated the rate of splitting as its frequency per flight duration (number of splitting events divided by the total seconds of flight).

### 2.2. Computational model of pigeon flocks

We extended our agent-based model of collective escape of homing pigeons, HoPE (Homing Pigeons Escape) [47] to study the emergence of splits and collective turns. HoPE is based on empirical data of homing pigeons [47]: it resembles real flocks in both individual behaviour (specifics of flying motion, speed and coordination rules among flock-mates [31,46,61,70]) as well as collective patterns [46,47]. Its detailed description according to the ODD (Overview, Design concepts, Detail) protocol [71] is given as an appendix in our electronic supplementary material.

HoPE consists of multiple pigeon-agents (referred to as *‘*pigeon-oids’, after Reynolds [21]) that coordinate with each other forming a flock and avoid one predator-agent (referred to as *‘*pred-oid’) that pursues them. Agents collect and update information about their environment (neighbours and predator) asynchronously at a given reaction rate, similar to previous realistic models of collective behaviour [24,72]. All model parameters are given in electronic supplementary material, table S3.

#### 2.2.1. Coordination and motion

Flocking in HoPE is based on the rules of attraction, alignment and avoidance among topological neighbours [24,30,43]. Coordination is controlled by the weighted sum of some social *‘*forces’ that represent these rules and has been adjusted to the specifics of flocking by pigeons [21,24]. Each pigeon-oid turns towards the centre of mass of its seven closest neighbours [30,33,47] and accelerates to stay close to them [31]. Alignment is represented by a force parallel to the average direction of all seven neighbours. Note that the effective number of interacting neighbours in the model can be decreased if there are not enough neighbours within a maximum distance in which pigeons are expected to coordinate [31,73], e.g. if two small sub-flocks with less than eight individuals are far away from each other. To avoid collisions, individuals turn away from their single closest neighbour (a rule proven to increase the resemblance of computational models to empirical data of bird flocks [25,43]). Alignment is set as the strongest of the coordination forces [31,46]. Each agent is also dragged towards its unique cruise speed (the more it deviates from it, the stronger this drag force becomes), in accordance to the empirical finding that pigeons have individualized preferred speeds from which they cannot deviate for a prolonged period [61].

#### 2.2.2. The predator

We modelled our predator-agent to resemble the motion of the RobotFalcon in the field experiment of Sankey *et al.* [46]. The pred-oid executes a hunting cycle of three distinct behaviours: (i) it follows the flock at a given distance (pursuit), (ii) it speeds up towards its closest pigeon-oid (attack), and then (iii) it moves far away from the flock (retreat). Each cycle lasts 1 min. *‘*Catches’ of prey by the predator are not modelled.

#### 2.2.3. Escape rules

In the original model [47], splitting of sub-flocks rarely emerges. To tackle this, we extended the individual escape behaviour by adding a discrete escape manoeuvre. Such manoeuvres have been identified in experiments of fish schools [74,75] and dunlin flocks [49] under attack, and have been used in a previous model of starling flocks to generate agitation waves [44].

Specifically, the discrete escape manoeuvre in our model involves a turn away from the heading of the predator [46] during multiple time steps. These turns are not fixed [59,60,76]; for each manoeuvre the turning angle and duration of turning are drawn independently from two gamma distributions (electronic supplementary material, table S3). We parametrized these distributions to our measures of individual turns in the empirical data (figure 2*b* and electronic supplementary material, figure S2). Following previous empirical findings, each flock member has a unique probability to manoeuvre based on (i) a unique baseline tendency to escape [62], and (ii) its distance to the predator (the closer it is to the pred-oid, the higher its probability to manoeuvre [46], electronic supplementary material, figure S13). While manoeuvring, the pigeon-oid does not coordinate with its neighbours.

Like in the original model, pigeon-oids are also steered away from the pred-oid by a continuous force of avoidance [26,47]. The direction of this force (escape direction) depends on the heading of the pred-oid, in line with the behaviour of real pigeons that turn away from the heading of the predator rather than its position [46]. When not manoeuvring, the motion of each pigeon-oid is controlled by the weighted average of this force away from the pred-oid and the forces of coordination among neighbours.

#### 2.2.4. Simulated data

We collected data from 1000 hunting cycles (total time 1000 min), simulating flocks of the same size as the empirical data (8, 10, 27 and 34 individuals). At each time step in the simulation, the larger flock was considered the *‘*main flock’ (defined as in the empirical data for consistency [46]). Results from all the simulations were combined into one dataset. For details on the simulations' set-up see the electronic supplementary material.

The focus of our analysis was to first determine which pattern of collective escape an *‘*initiator’ (the individual performing an escape manoeuvre) causes, and subsequently to examine how the relative frequency of these patterns depends on the characteristics of the initiator. In detail, we study the initiator's position in the flock based on its *‘*centrality’, i.e. an individual's spatial position relative to all its flock-mates (for the exact definition see electronic supplementary material S1.2). We further make the centrality of individuals comparable between members of small and large flocks by dividing it by the average centrality of all individuals in small and large flocks, respectively. Individuals with centrality less than the median of the distribution across all individuals and flights are characterized as *‘*central’, opposite to individuals with higher centrality labelled as *‘*edge’. Secondly, the direction of escape is labelled as *‘*inwards’ or *‘*outwards’ depending on whether the escape turn is towards the flock's centre (relative to the individual's position) or away from it. We measured these initiator's traits at the beginning of each escape manoeuvre. Thirdly, we get the initiator's angular velocity based on the total change of heading during the manoeuvre divided by its duration, and we label as ‘low’ and ‘high’ turns with angular velocities higher and lower than the median, respectively. We studied the rate of the two collective patterns as the frequency of their appearance relative to the total number of escape manoeuvres. We confined ourselves to patterns generated in the main flock.

### 2.3. Analysis of individual motion

To test the validity of our theoretical findings for real flocks of pigeons, we studied in the empirical data the association between the turning characteristics of individuals and the collective pattern when attacked by the RobotFalcon [46]. Using the same method as for the flocks' turning motion (see §2.1.1), we measured the turning of individual flock members. We distinguished two types of turns: (i) *‘*collective turns’, during which the individual remains part of the flock, and (ii) *‘*splitting turns’, during which the individual splits off from it. Note that here we focus on splitting individuals and not on identifying initiators of collective turns; we treat each individual's turn while being part of a flock as absence of splitting from it. As a measurement of centrality, we use the distance of each individual to the centre of the flock divided by the mean centrality of small or large flocks (as in the analysis of the simulated data). We constructed generalized linear mixed models (GLMMs) on the binomial variable ‘turning type’ (with values one of the two turns defined above, collective or splitting), and with fixed variables being the angular velocity and centrality of each individual at the beginning of each turn [77,78]. Given that the data include several instances of an individual's motion and that members of the same flock interact with each other, we added the unique flight number and pigeon identity as random effects in our models. For details, see our electronic supplementary material.

### 2.4. Software

Our computational model is built with the programming language C++ 17, using OpenGL for visualizing the simulations. All data analyses and statistical modelling were performed in R (v. 3.6) [79] and all graphs were generated using the ‘ggplot2’ package [80].

## 3. Results

### 3.1. Collective escape of pigeons

We identified two patterns of collective escape in the empirical data: the collective turn and the split (figure 2). In detail, across 27 flights with the RobotFalcon attacking the flock, we measured 155 collective turns of flocks and 65 events of flocks splitting (35 solitary departures and 30 splits of sub-flocks). Note that both patterns appear across varying distances to the predator (electronic supplementary material, figure S3).

The rate of each pattern is significantly higher in the presence of the RobotFalcon than in its absence (table 1). Small flocks in particular do not split in the absence of the predator (electronic supplementary material, table S2). The proportion of time spent in collective turning and the turning angle per second of flight are significantly larger in flocks under attack than unthreatened ones. The threshold of angular velocity for distinguishing a turn from straight flight does not affect our conclusions (electronic supplementary material, table S1).

**Table 1.** Measurements of collective turning and splitting-off in the empirical data of flights with the predator (*n* = 27) and without it (*n* = 16) (***p-value < 0.001, **p-value < 0.01, *p-value < 0.05). *N* is the total frequency of each pattern. We do not statistically compare these absolute frequencies because of the difference in sample size. Rates correspond to the across-flights median of number of events divided by flight duration. We use the median because of the highly skewed shape of the distributions. Statistical significance is based on two-sample Kolmogorov–Smirnov tests between the measurements distributions in flights with and without the predator (flight-time spent turning: *D* = 0.488, *p*-value = 0.01; angle per second: *D* = 0.431, *p*-value = 0.03; turning rate: *D* = 0.602, *p*-value < 0.001; splitting rate: *D* = 0.454, *p*-value = 0.03).

collective pattern	measurement	without predator	with predator	*p*-value
collective turn	*N*	50	155	
	flight-time spent turning (%)	30	55	**
	angle per second (° s^−1^)	9	15	*
	turning rate (turns s^−1^)	0.08	0.15	***
split	*N*	33	65	
	splitting rate (splits s^−1^)	0	0.024	*

### 3.2. Collective escape in the computational model

The flocking model HoPE resembles real flocks of pigeons in several characteristics, namely their distributions of individual speed, nearest neighbour distance and relative position of neighbours ([47], electronic supplementary material, figure S4). Both splitting and collective turning emerge from the uncoordinated escape manoeuvre (figure 2, electronic supplementary material, videos S2 and S3). When the majority of the flock follows the initiator into a turn, a collective turn takes place. When none of the flock members follows, the initiator splits off from the group alone. If fewer than half of the flock follows the initiator, a sub-flock splits off (which constitutes approximately 10% of all splits in our simulated data). According to rules of information propagation through a group [36,66,81], we hypothesized that more collective turns will be initiated by individuals that are turning slowly (with low angular velocity) and inwards, and are positioned close to the centre of their flock.

**Figure 3.**
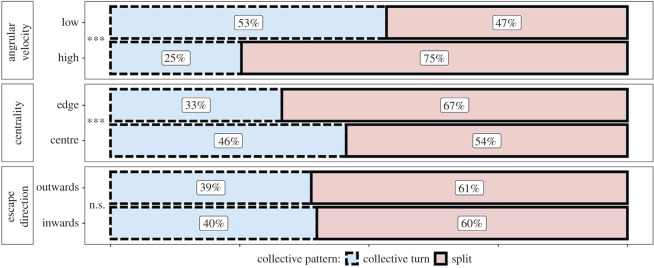
The effect of a manoeuvring individual (turn initiator) on the rates of collective escape patterns in the simulation (***p-value < 0.001, n.s.: *p*-value > 0.05). Escape direction does not significantly affect the relative frequency of splits and collective turns (Pearson's chi-squared test: *χ*^2^ = 0.74, d.f. = 1, *p*-value = 0.39). The effect of the angular velocity and centrality (position in the flock) of an individual is significant (Pearson's chi-squared tests, centrality: *χ*^2^ = 105.54, d.f. = 1, *p*-value < 0.001; angular velocity: *χ*^2^ = 539.34, d.f. = 1, *p*-value < 0.001).

High angular velocity of an escape manoeuvre (higher than the median angular velocity, referred to as ‘sharp turn’) leads to a higher probability to split off from the group (a 28% increase relative to manoeuvres of low angular velocity, figure 3). Manoeuvres by individuals closer to the flock's centre lead to collective turns more often (approx. 10% increase) than manoeuvres by agents at the edge of the flock (centrality distributions differ significantly between splits and collective turns, according to a two-sided Kolmogorov–Smirnov test: *D* = 0.15, *p*-value < 0.001). The relative frequency of collective turns and splits does not significantly differ between outward and inward manoeuvres (see caption of figure 3). The effects remain for the unique combinations of these characteristics (see electronic supplementary material, figure S6), for instance, edge individuals with high angular velocity have higher probability of splitting than centre individuals with high angular velocity. Guided by our model, we test in the empirical data whether the sharpness of an individual's turn (its angular velocity) and its position in the flock (proximity to the flock's centre) affect the emergence of splits or collective turns.

### 3.3. From individual turns to collective patterns

**Figure 4.**
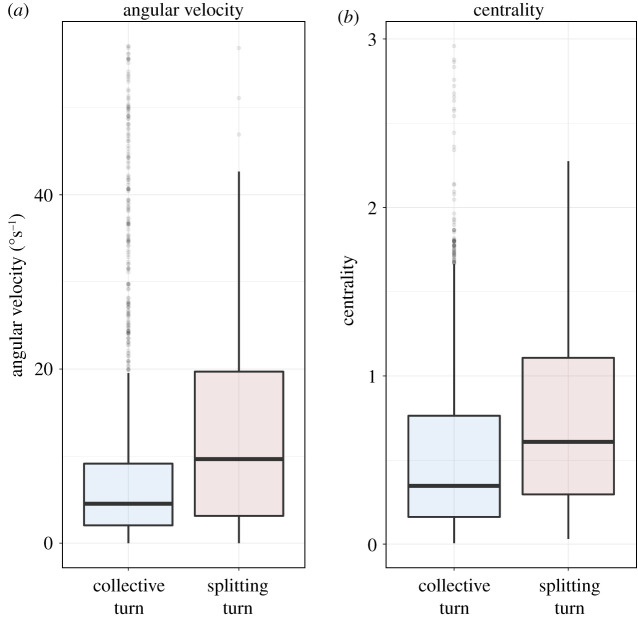
The distribution of (*a*) angular velocity and (*b*) centrality at the beginning of all individual turns in the empirical data of pigeons under attack by the RobotFalcon, across the two turning types (splitting and collectively turning). Each box is drawn from the first to the third quantile of each distribution and the horizontal line through it shows the median. Points represent outliers over the 90% quantile. Points above the 95% of the distribution are omitted for visualization (see electronic supplementary material, figure S7 for the full distributions, electronic supplementary material, figure S10 for a comparison with the simulated data and electronic supplementary material, figure S11 for the relation of the angular velocity and centrality in both datasets).

We tested in the empirical data the hypothesis based on our modelling results (figure 3): individuals split off more often when they are turning more sharply and are positioned more towards the edge of their flock. We confirm that high angular velocity associates more with splitting than collectively turning (GLMM: regression coefficient *β* = −1.27, s.e. = 0.32, *z*-value = −4 and *p* < 0.001; figure 4*a*). Centrality does not show a significant effect on turning type (GLMM: *β* = 0.08, s.e. = 0.12, *z*-value = 0.68 and *p* = 0.5). However, if outliers above the 90% quantile of distance to the centre of the flock are filtered out (electronic supplementary material, figure S7), centrality is positively associated with splitting (GLMM: *β* = −0.66, s.e. = 0.26, *z*-value = −2.5 and *p* = 0.01; figure 4*b*), especially in close distances to the predator (less than 100 m, GLMM: *β* = −0.9, s.e. = 0.3, *z*-value = −3.03 and *p* = 0.002). For the validity of our fitted models see electronic supplementary material, figure S8, S9.

## 4. Discussion

To gain insight in what underlies patterns of collective escape in homing pigeons, we combined GPS data of flocks under attack by the RobotFalcon [46] with a species-specific model based on self-organization [47]. We analysed the tracks of flocking pigeons and identified two patterns of collective escape: collective turns and splits (of sub-flocks or singletons), even at large distances from the predator. So far, collective turns of the whole flock have been overlooked as a pattern of collective escape in previous studies [2,26]. In our computational model of collective motion (adjusted to pigeon flocks, [47]) both patterns of collective escape arise when a single group member (the initiator) performs a discrete, uncoordinated and stochastic manoeuvre by turning away from the predator (electronic supplementary material, videos S2 and S3). Whether splitting or collective turning emerges depends mainly on the angular velocity of the initiator and its position relative to the flock's centre, not only in the model but also in the empirical data.

Several factors may increase the angular velocity of a bird's turn in nature; first, past and present predatory risk [82], closer distances to the predator [46,58,83] and increased level of alertness of the group [55,82]. Secondly, the predator's behaviour [74,84–86]. A faster predator may cause prey to turn more sharply [13,86]. This, however, may be unnecessary if a wider turn is less **‘**costly’ (energetically cheaper or easier) to perform and has been proven successful in the past [82]. Prey may also react differently to the direction of a predator's attack (whether it approaches the flock from the side or from behind) [84,85,87]. Thirdly, the intensity of an escape (as indicated by the speed, frequency or amplitude of motion) may be prone to social enhancement by surrounding flocks [10] or dependent on the dominance of the turning individual (as shown for crayfish, where dominant individuals are more likely than subordinates to execute reflexive and sharp escape responses [88]). In our model, we showed that manoeuvres of high angular velocity lead to more splits than collective turns**.**

The occurrence of a split or a collective turn may also depend on how quickly a turn propagates through the group. The speed of this propagation (information transfer concerning the turning direction) may be heavily affected by the specifics of coordination. These specifics differ across species, for instance whether interactions are topological (with fixed number of closest neighbours) or metric (with all individuals within a specific range), and the relative importance of alignment, attraction and repulsion [30–33,36,37,46]. For species in which interactions among flock members are biased (e.g. homing pigeons and jackdaws), how information propagates through the group may also depend on leadership dynamics [89], mating bonds [90] and individual differences in experience [73] and fitness [91]. The direction from which the turn starts may also play an important role in the speed of information transfer.

The position of initiators of collective turns in airborne flocks varies across bird species and ecological contexts. During roosting, mobbing and transit flights [36,37,65], starlings that initiate turns are located at the periphery [65], whereas in jackdaws these initiators are mostly at the front of the flock [36]. The initiation of collective turns has not yet been studied during collective escape from an avian predator. In our model, group members positioned more centrally in a flock initiate collective turns more frequently than individuals at the edge. This variation may be due to across-species differences in the size and shape of their flocks; individuals in the centre of small and flat flocks [92] can probably turn more freely than in flocks that are three-dimensional, large and dense [93,94].

There may be several reasons that pigeon flocks turn away from a predator at large distances to it. Pigeons often form small flocks with low density. Therefore, their flocks may not confuse the predator enough to decrease its hunting success (no confusion effect [95]), and individuals may have higher probability of getting caught than when they are in large flocks (no selfish herd [46]). According to our model, early collective escape of a flock may be the result of protean movement of its members. Thus, given the variability of the emerging collective turns and splits, the escape path of the group may become less predictable [59,60,76], counteracting anticipation-based hunting strategies of the predator [58,67,96].

The role of uncoordinated escape by group members during collective motion has not yet been studied. Uncoordinated turning by an initiator, as implemented in our model, causes a collective turn to be larger, given that it is not a compromise between the headings of the initiator and its neighbours [52,97]. This may be more effective for avoiding the predator. Thus, an uncoordinated escape may be adaptive for the whole group and actually reflect a ‘cooperative’ [98], instead of a ‘selfish’ [52,99], act. Along the same lines, referring to an individual's split from the group as a ‘decision’ to leave should be done with caution [46] since our findings show that a splitting event is not decided at the individual level, but instead results from the behaviour of the followers.

The consequences of a split for the survival of the prey and the success of the predator may depend on the strategy of both. The success of hunting by predators is higher when the flock is smaller and when attacking singletons, rather than flocks [11,100]. From the perspective of the prey, the danger of splitting from the group when performing a sharp, evasive turn may be balanced by the prey's probability of getting rid of the predator by outperforming it [96]. A predator may, however, catch a sharply manoeuvrable singleton through stooping at high speed, because stooping optimizes the predators aerodynamic forces and roll agility [86]. Whether the number of initiators of an escape manoeuvre influences the type of collective escape pattern or the prey's survival remains to be studied.

In pigeon flocks, we identified fewer patterns of collective escape (splits and collective turns) than have been described for other species [2,49]. This may have several reasons. First, more complex patterns, such as vacuoles and agitation waves may require larger flock sizes [2,26]. Secondly, the large body size of pigeons and their low manoeuvrability [86,101,102] may prevent them from executing the sharp turns involved in a flash expansion [26,103]. Thirdly, the slower attack of the artificial predator (RobotFalcon) in comparison to a real falcon [2] may prevent some patterns that depend on the quick increase in the proximity to the predator to emerge [84,87,97]. Fourthly, the strong alignment of heading of homing pigeons [31,46] may support collective turns and counteract patterns with large divergence in the heading of group members, such as the fountain [53].

The rules of individual escape that were used in previous models did not suffice to produce splits at large distances to the predator and collective turns [26,52]. Most previous models assume that group members share a single escape behaviour: a tendency to turn away from the position or heading of the approaching predator [26,47,52]. From this rule, splits emerge mostly when the predator gets close to the group and often passes through it (causing sub-flocks to turn away from the predator in opposite directions), while collective turns do not arise. In models with only a discrete escape reaction, individuals need an additional rule of ‘copying’ any manoeuvre performed by their neighbours in order for an escape wave to emerge [44,55,104,105]. This additional rule of coordination counteracts splitting. In our model, we combine the continuous tendency to avoid the predator and discrete escape manoeuvres. From only these two rules, collective turns emerge instead of splits, if group members follow an initiator through the basic rules of coordination (alignment and attraction) and their own tendency to turn away from the predator. Guided by empirical data, we give each group member a varying, unique probability to perform an escape manoeuvre. With our new probabilistic approach, collective patterns arise as precautious reactions to the predator, rather than ‘panic-like’ attempts to survive when the predator is near.

Future work on empirical data of airborne flocks under attack should focus on individual escape reactions, the number and relative position of initiators of turns and information transfer in relation to flock shape and size [55,90,106]. Moreover, quantitative comparisons of patterns of collective escape across species, combined with biologically relevant computational models, can significantly deepen our understanding of what rules underlie these complex patterns.

## Data accessibility

The agent-based model (HoPE) is available on GitHub: https://github.com/marinapapa/a-new-HoPE-model and the version used for this research work has been archived within the Zenodo repository: https://doi.org/10.5281/zenodo.5889123 [107]. Data and relevant code for this research work are stored in GitHub: https://github.com/marinapapa/PigeonsCollectiveEscape and have been archived within the Zenodo repository: https://doi.org/10.5281/zenodo.5889098 [108]. The raw data are deposited in the Dryad Digital Repository: https://doi.org/10.5061/dryad.905qfttkc [109].
